# 3D Nanoparticle Tracking Inside the Silver Nanofluid

**DOI:** 10.3390/nano10020397

**Published:** 2020-02-24

**Authors:** Saeid Vafaei

**Affiliations:** Mechanical Engineering Department, Bradley University, Peoria, IL 61625, USA; svafaei@fsmail.bradley.edu

**Keywords:** nanoparticle, nanofluid, tracking nanoparticles three-dimensionally (3D)

## Abstract

Movement of nanoparticle was investigated at the vicinity of silver nanofluid by using a microscope equipped with 100X lens. It was observed that silver nanoparticles were constantly moving inside the nanofluid for the first time. To explore the silver nanoparticle movement, the silver nanofluid was mixed with fluorescent nanoparticles. The coated nanoparticles were tracked three-dimensionally using a Delta Vision Elite inverted optical microscope. It was found that Marangoni flow was a possible reason of the nanoparticle movement which was generated by a gradient of the surface tension at the vicinity of the triple line. A gradient of the surface tension was formed by the segregation of the surfactant from the base liquid at the vicinity of the triple line. The surfactant was separated from the base liquid inside the triple region, since they have different affinities for the substrate. It was also shown that ring phenomenon took place when nanoparticle movement was weak or negligible.

## 1. Introduction

Silver nanofluid can be used to print electronic tracks three-dimensionally (3D). Silver nanoparticles are mixed with the base liquid to produce the high concentration silver nanofluid. Generally, the silver nanofluid is mixed with surfactant to prevent agglomeration and deposition of nanoparticles. The uniform distribution of silver nanoparticles inside the base liquid plays a significant role on homogeneity of printed tracks. The quality and resolution of 3D printed tracks depend on nanoparticle movement in the triple region during the drying process. Therefore, it is necessary to understand the mechanisms of possible nanoparticle movement inside the nanofluid.

**Figure 1:**
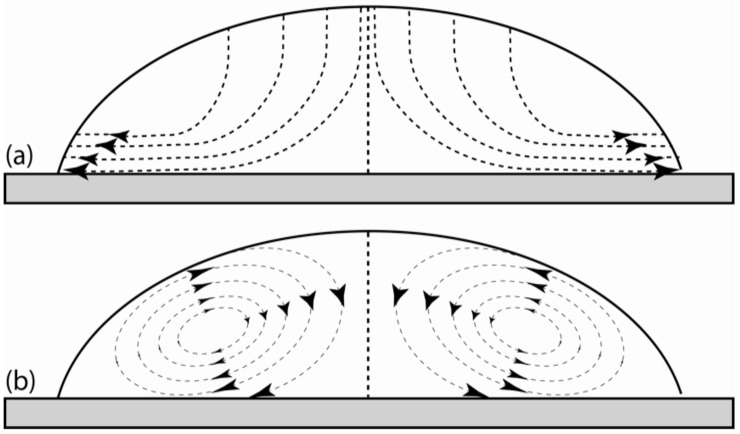
Schematic of (**a**) outward capillary flow during the drying process, and (**b**) Marangoni flow (circulatory fluid flow) inside a droplet.

Marangoni flow is a possible reason for flow motion and consequently nanoparticle movement inside the nanofluid. The Marangoni effect can be caused by a surface tension gradient which may be related to temperature variation [1] along the free liquid surface (thermal effect). As a result of a surface tension gradient, the liquid moves at the liquid–gas interface, from a lower surface tension point to a higher surface tension point. Marangoni flow during water droplet evaporation has been observed by using fluorescent nanoparticles. The non-uniform evaporation of liquid [1] has been reported to cause a temperature gradient, and consequently a surface tension gradient along the liquid–gas interface which drives the flow inside the liquid. When the evaporation rate is relatively significant, the high latent heat of liquid [2] can be a reason for a temperature gradient [3] at the liquid–gas interface in the vicinity of the triple line. A temperature gradient at the liquid–gas interface might be produced because of the long conduction distance between the triple line and apex [2,4] which can generate a surface tension gradient. The temperature variation along the liquid–gas interface might not always be monotonic, and there is a possibility for a stagnation point for a temperature and surface tension gradient [1]. It was also expressed that Marangoni flow can be suppressed by adding surfactant into the base liquid or by being contaminated with surfactant on the substrate [5]. Introducing surfactant into the base liquid has the potential to reduce a surface tension and consequently suppress a surface tension gradient. However, the suppression level of Marangoni flow depends on physical properties of solid, gas, and liquid. The enhancement of Marangoni flow has been reported while the nanofluid droplet was dried in an ethanol vapor environment. Marangoni flow during the drying period in ethanol vapor environments allowed a significant inward flow of particles from the triple region, and consequently enhanced the uniform nanoparticle deposition across the substrate [6] and reversed the ring deposition of nanoparticles at the triple region. During the evaporation of a coffee droplet on substrate, it was observed that dispersed solids migrated toward the triple line, forming a solid ring which is called a ring phenomenon. The strong liquid evaporation in the triple region drowned the liquid from the interior as a result of the capillary flow, which resulted in an outward capillary flow that carried dispersed particles to the edge of the triple line and formed a ring pattern at the vicinity of the triple line. It has also been observed that the shape and thickness of the deposited nanoparticles (ring deposition) may be controlled by the evaporation rate [4,7]. It has been shown that particle density and relative humidity influence the interfacial entrapment and cross-sectional distribution of nanoparticles [8]. On the contrary, it was observed that Marangoni flow reversed the ring deposition and spread the nanoparticles more into the nanofluid bulk [2] which is able to prevent the ring deposition of nanoparticles. In general, Marangoni flow has a great potential to form and control the pattern and uniformity of deposited nanoparticles on the substrate at the vicinity of the triple line. The final pattern of deposited nanoparticles depends on competition between outward capillary flow [4,9] and Marangoni flow, which determines the shape of deposited nanoparticles to be either ring pattern, uniform, or homogeneous bumps [9]. For instance, the fingering pattern was observed to be inside bi-dispersed colloid droplets (the mixture of nanometer and micrometer particles) during the evaporation while inward Marangoni flow was overwhelmed by an outward capillary flow. In the first step, the smaller and bigger particles respectively deposited in outer and inner rings. Then, the finger pattern was formed, and the inner ring width was increased by an outward capillary flow [10]. This sort of circulatory fluid flow inside a drying droplet has the potential to separate the particles with different sizes [11]. The outward capillary and Marangoni flow inside a droplet in the drying period can be seen in Figure 1. Many phenomena can be affected by Marangoni flow such as melting [12], fluid dynamics, and heat transfer around a bubble [13,14,15,16,17].

The circulatory fluid flow inside a drying nanofluid droplet might be triggered by sequential pinning and de-pinning cycles of the triple line, which can be controlled by nanoparticle concentration and surface tension of nanofluid [11]. De-pinning of the triple line might be related to the enhancement of a solid–gas surface tension due to the nanoparticle deposition by time. After de-pinning, the triple line recedes until it reaches another equilibrium position. The movement and displacement of the triple line mainly depends on the force balance at the triple line between liquid–gas, *σ_lg_* solid–gas, *σ_sg_* and solid–liquid, *σ_sl_*, and surface tensions [18].

The concentration and characterization of nanoparticles have a great potential to change the liquid–gas surface tension, [6,19] and solid surface tensions, *σ_sg_* − *σ_sl_* of nanofluids [18,20]. The liquid–gas surface tension of bismuth telluride nanofluids (2.5 nm, 10.4 nm) has been reported to be decreased with a concentration of nanoparticles. As the nanofluid concentration increased, the liquid–gas surface tension increased. A similar trend has also been reported for a solid surface tension [18,20]. A reduction of more than 50% of the liquid–gas surface tension has been observed for bismuth telluride nanoparticle (2.5 nm) suspension. More nanoparticles were driven to the liquid–gas interface region as concentration of bismuth telluride nanofluid increased [19]. The nanoparticles were bounded at the interface [21]. In contrast, the effects of nanoparticles on the liquid–gas surface tension of aluminum–ethanol [22] and alumina–water [23] nanofluids have been reported to be weak. Generally, the characteristics and concentration of nanoparticles, base liquid materials, and concentration of possible surfactants have significant effects on liquid–gas and solid surface tensions [18,20].

In general, the nanoparticle motion was observed in nanofluid with low concentrations because of Marangoni flow. Marangoni flow can be generated because of variation of the liquid–gas surface tension from one point to another, as a result of a temperature gradient which can be caused by liquid evaporation at the triple line (thermal effect). In this research, the nanoparticle motion was investigated (a) when evaporation of the base liquid was negligible, and (b) when the base liquid was water and evaporation of the base liquid was not negligible. The purpose of this study is to demonstrate the nanoparticle movement and explain the possible mechanisms of nanoparticle movement inside the nanofluid.

## 2. Experimental Setup

**Figure 2:**
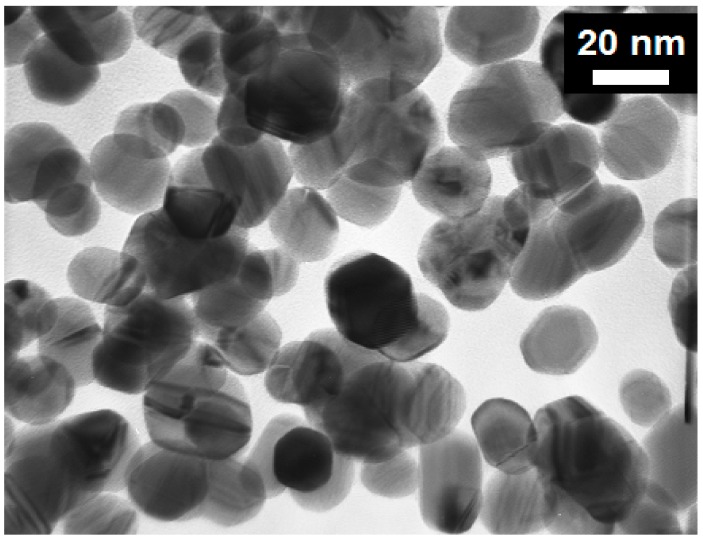
Transmission electron microscopy (TEM) image of silver nanoparticles.

Silver nanoparticles (30–40 nm) were mixed with triethylene glycol monoethyl ether (TGME) to create silver nanofluids with 38.853 w%. Transmission electron microscopy (TEM) image of silver nanoparticles can be seen in Figure 2.

FluoSpheres, carboxylate-modified microspheres, 100 nm, and yellow–green fluorescent were mixed with silver nanofluid to study the nanoparticle movement inside the silver nanofluid. The movement of fluorescent nanoparticles was tracked inside silver nanofluid by using a Delta Vision Elite inverted optical microscope. The weight ratio of fluorescent nanoparticles inside the silver nanofluid was 1.0308 × 10^−5^. Image processing was performed on a Delta Vision Elite inverted optical microscope (Applied Precision), built around a stand (IX71; Olympus) equipped with a 100X 1.4 NA objective lens, Sedat QUAD (Chroma) Polychroic and an EMCCD camera (Photometrics). The fluorescent sample was excited by 520–565 nm light from a solid-state light source (Lumencore). Emitted light was collected through a 594/45 band-pass filter. Optical sections (4–5), 0.1 µm apart were scanned and recorded every 50 milliseconds. To track the maximum number of nanoparticles, the thickness and number of optical sections were optimized. A Volocity software was used to track fluorescent nanoparticles inside the silver nanofluid three-dimensionally. To validate the nanoparticle tracking by a Delta Vision Elite inverted optical microscope, similar tracking was conducted during the evaporation of a water nanofluid droplet (the mixture of fluorescent nanoparticles and water).

## 3. Results and Discussion

**Figure 3:**
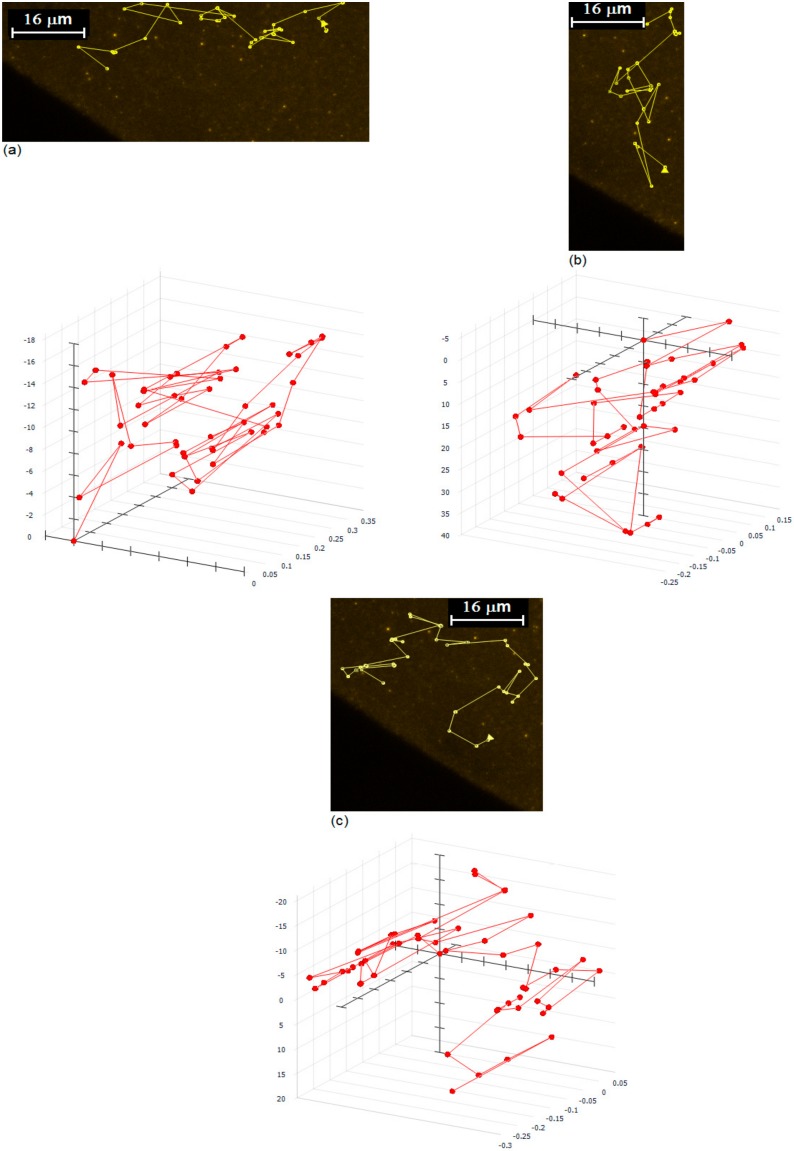
Two- (2D) and three-dimensional (3D) tracking of nanoparticles inside silver nanofluids. Dimensions of *x*, *y*, and *z* are in micrometer.

The layering phenomenon of silver nanofluid at the triple region was investigated, using an Environmental Scanning Electron Microscope (ESEM) by the author [24]. During the experiment, it was observed that nanoparticles were moving at the end of the triple region where there was a tiny layer of nanoparticles on substrate. This observation initiated further investigations of nanoparticle movement inside the silver nanofluid. The movement of silver nanoparticles inside the nanofluid was investigated, using an optical microscope equipped with 100X lens. To investigate the nanoparticle movement, silver nanofluids were mixed with nanoparticles, coated with fluorescent materials. The movement of nanoparticles was observed to be very complicated and complex. The strength of Marangoni effect and concentration of the silver nanofluid have key roles on complexity of nanoparticle movement. The possibility of collusions and redirections of particles increases with a concentration of nanoparticles, and the possibility of redirection of nanoparticles due to collusions increases with reduction of Marangoni effect. Practically, only a strong Marangoni effect can produce a circulatory fluid flow as shown in Figure 1a. Figure 3 shows the typical tracking of fluorescent nanoparticles inside the silver nanofluid. It was observed that (a) nanoparticles moved from the triple line toward bulk (outward fluid flow), (b) nanoparticles moved from bulk toward the triple line (inward fluid flow), and (c) nanoparticles moved from the triple line toward bulk and back to the triple line (circling fluid flow).

**Figure 4:**
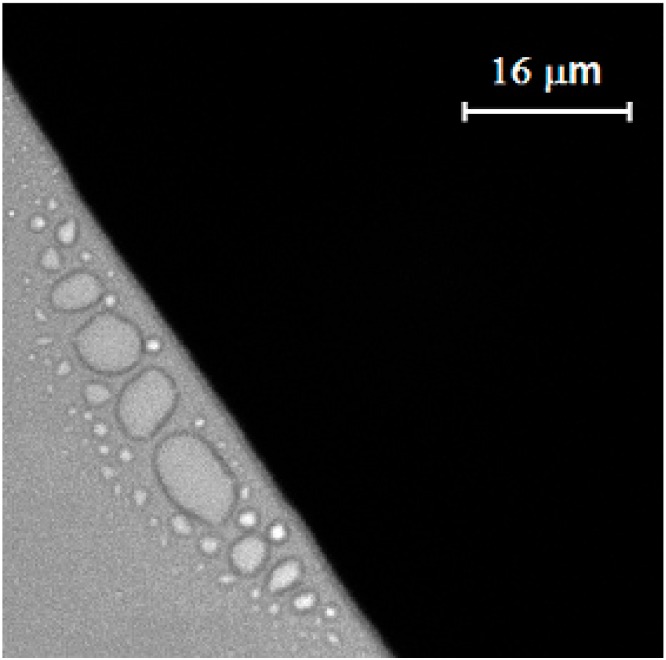
Separation of surfactant from the base liquid at the vicinity of the silver nanofluid triple line was observed on a glass substrate by microscope.

Evaporation of the silver nanofluid in ambient temperature is negligible, since the boiling temperature of the base liquid (triethylene glycol monoethyl ether) is about 255 °C, so the temperature gradient caused by nanofluid evaporation at the vicinity of the triple line may not be a reason for nanoparticle movement. The Brownian effect may also not be a reason due to directional movement of nanoparticles. It was found that surfactant was separated from the base liquid on the solid surface at the vicinity of the triple line in the triple region, because of different affinities of the base liquid and surfactant for solid substrate which was generated from a liquid–gas surface tension gradient in and out of the triple region. As a result of a liquid–gas surface tension gradient, the base liquid moved from a low surface tension point toward a high surface tension point and created a movement inside the nanofluid. Figure 4 shows the separation of surfactant from the base liquid at the vicinity of the triple line on the glass substrate. The surfactant was separated from the base liquid, because of a higher affinity of surfactant for glass. It was found that concentration of surfactant has significant effects on the liquid–gas surface tension [25].

**Figure 5:**
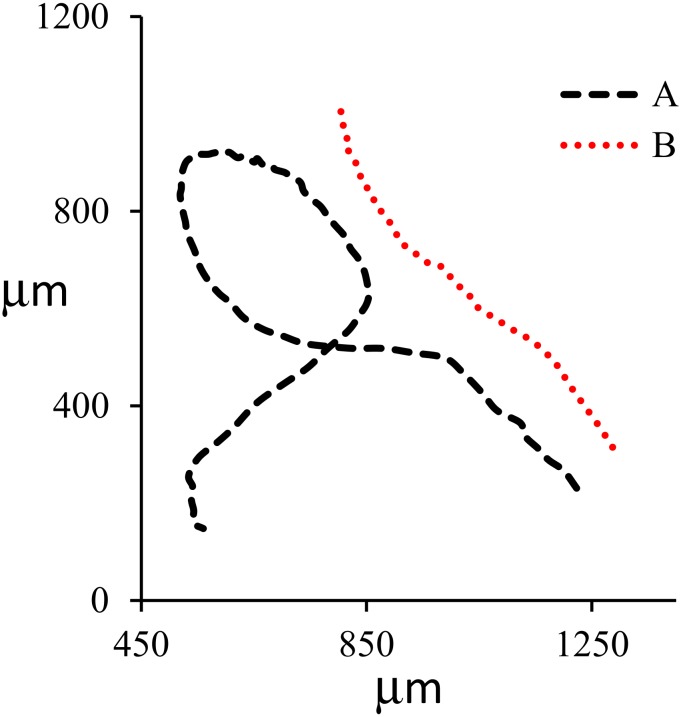
Typical 2D pattern of nanoparticle movement. Nanoparticles were circling (**A**) and moving outward (**B**).

**Figure 6:**
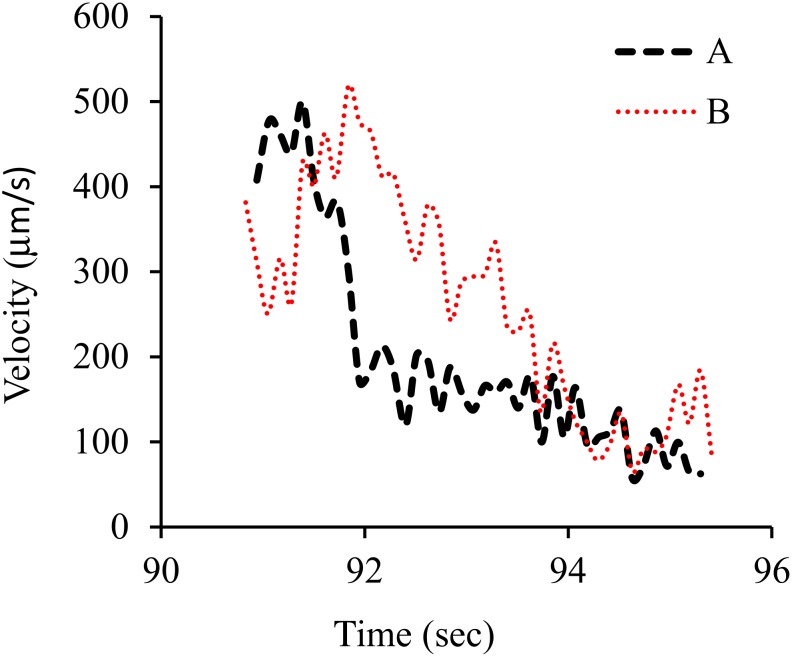
Variation in velocity of nanoparticles as a function of time. Nanoparticles were circling (**A**) and moving outward (**B**).

Similarly, evaporation of a drop of gold–water nanofluid was observed by a microscope equipped with a 100X lens. A Volocity software was used to track the gold nanoparticles inside nanofluid. Gold nanoparticles were 5 nm and spherical. The gold nanoparticles were tracked at the vicinity of the triple line two-dimensionally (2D). It was observed that some nanoparticles moved toward the liquid bulk, some toward the triple line, and some toward the bulk and then returned toward the triple line. Figure 5; Figure 6 show the pattern of nanoparticle movement and the velocity of nanoparticle movement, respectively. Nanoparticles were circling in case (A) and moving outward in case of (B). Average velocity was 260 μm/s in case (A) and 199 μm/s in case (B).

**Figure 7:**
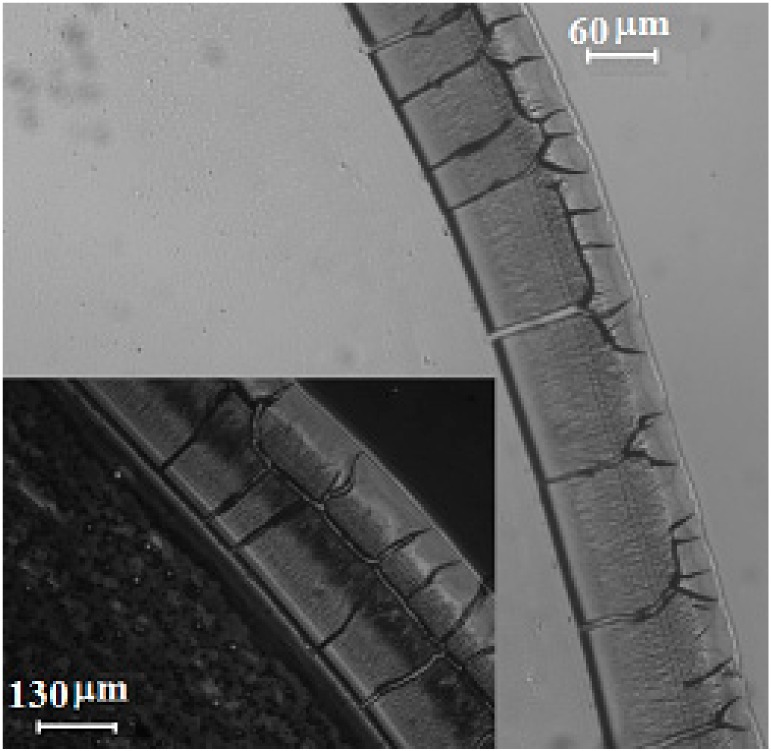
Ring phenomenon caused by evaporation of mixture of water and fluorescent nanoparticles on glass substrate.

Figure 7 shows the ring phenomenon during the evaporation process of a nanofluid droplet on glass substrate. The nanofluid was a mixture of water and fluorescent nanoparticles. The strong liquid evaporation in the triple region drowned the liquid from interior as a result of a capillary flow, which resulted in an outward capillary flow that carried dispersed particles to the edge of the triple line and formed a ring pattern at the vicinity of the triple line. Tracking of coated nanoparticles was examined and validated by observing nanoparticle movement during the evaporation of mixture of water and fluorescent nanoparticles, using a Delta Vision Elite inverted optical microscope. The movement of nanoparticles and the formation of the ring pattern at the vicinity of the triple line during the evaporation are a well-known phenomenon reported by several researchers [4,7]. Interestingly, the ring phenomenon was not observed in case of silver nanofluid on a glass substrate, which might be related to nanoparticle movement as a result of Marangoni flow inside silver nanofluid.

## 4. Conclusions

The motion of nanoparticles inside the silver nanofluid was investigated using an optical microscope equipped with a 100X lens. The nanoparticle movement was observed inside the silver nanofluid at the vicinity of the triple line because of a surface tension gradient in and out of the triple region. As a result of the different affinity of the base liquid and surfactant for the solid substrate, the surfactant was separated from the base liquid at the vicinity of the triple line which generated a surface tension gradient in and out of the triple region. The surface tension gradient at the vicinity of triple line generated a Marangoni flow, and consequently nanoparticle movement inside the silver nanofluid at the vicinity of the triple line. Marangoni flow was observed to be strong enough to make the nanoparticle collusion, but also weak enough not to produce a complete circulatory fluid flow. Practically, directional nanoparticle collusions were observed as a result of a weak Marangoni flow in high concentrated silver nanofluid (see Figure 3).

In addition, movement of nanoparticles was observed during the evaporation of gold–water nanofluid droplet on the similar glass, two-dimensionally (2D). Three kinds of nanoparticle movements were observed at the vicinity of the triple line such as circling and inward and outward flows. The nanoparticle motion was because of Marangoni flow which was generated as a result of thermal effect.

Similarly, movement of nanoparticles was observed during the evaporation of silver nanofluid droplet. Drying the mixture of fluorescent nanoparticles with silver nanofluid was compared with that of water. The ring phenomenon was not observed in case of drying the mixture of fluorescent nanoparticles and silver nanofluid because of nanoparticle movement. On the contrary, a solid ring pattern was observed while the mixture of water and fluorescent nanoparticles was dried on the same glass substrate.

Marangoni flow has a great potential to form and control the pattern and uniformity of deposited nanoparticles on the substrate at the vicinity of the triple line. The final pattern of deposited nanoparticles depends on competition between outward capillary flow and Marangoni flow.

## Abbreviations

Greek Symbols	
*σ_lg_*	liquid–gas surface tension (N/m)
*σ_sg_*	solid–gas surface tension (N/m)
*σ_sl_*	solid–liquid surface tension (N/m)

## References

[1] Xu X., Luo J. (2007). Marangoni flow in an evaporating water droplet. Appl. Phys. Lett..

[2] Hu H., Larson R.G. (2006). Marangoni effect reverses coffee ring depositions. J. Phys. Chem. B.

[3] Steinchen A., Sefiane K.J. (2005). Self-organised Marangoni motion at evaporating drops or in capillary menisci-Thermohydrodynamical model. J. Non-Equilib. Thermodyn..

[4] Deegan R.D., Bakajin O., Dupont T.F., Huber G., Nagel S., Witten T.A. (2000). Contact line deposits in an evaporating drop. Phys. Rev. E.

[5] Hu H., Larson R.G. (2005). Analysis of the effects of Marangoni stresses on the microflow in an evaporating sessile droplet. Langmuir.

[6] Majumder M., Clint S., Rendall C.S., Eukel J.A., Wang J.Y.L., Behabtu N., Cary L., Pint C.L., Liu T.Y., Orbaek A.W. (2012). Overcoming the coffee-stain effect by compositional Marangoni-flow-assisted drop-drying. J. Phys. Chem. B.

[7] Deegan R.D., Bakajin O., Dupont T.F., Huber G., Nagel S.R., Witten T.A. (1997). Capillary flow as the cause of ring stains from dried liquid drops. Nature.

[8] Trantum J.R., Eagleton Z.E., Patil C.A., Tucker-Schwartz J.M., Baglia M.L., Skala M.C., Haselton F.R. (2013). Cross-sectional tracking of particle motion in evaporating drops: Flow fields and interfacial accumulation. Langmuir.

[9] Bhardwaj R., Fang X., Attinger D. (2009). Pattern formation during the evaporation of a colloidal nanoliter drop: A numerical and experimental study. New J. Phys..

[10] Weon B.M., Je J.H. (2013). Fingering inside the coffee ring. Phys. Rev. E.

[11] Li H., Fowler N., Struck C., Sivasankar S. (2011). Flow triggered by instabilities at the contact line of a drop containing nanoparticles. Soft Matter.

[12] Shuja S.Z. (2014). Laser heating of tungsten carbide-coated steel surface: Influence of coating thickness on temperature field and melt depth. Heat Transfer Eng..

[13] Radulescu C., Robinson A.J. (2010). Mixed convective heat transfer due to forced and thermocapillary flow around bubbles in a miniature channel: A 2D numerical study. Heat Transfer Eng..

[14] Radulescu C. (2012). Mixed thermocapillary and forced convection heat transfer around a hemispherical bubble in a miniature channel: A 3D numerical study. Heat Transfer Eng..

[15] O’Shaughnessy S.M., Robinson A.J. (2009). The influence of the magnitude of gravitational acceleration on Marangoni convection about an isolated bubble under a heated wall. Heat Transfer Eng..

[16] Qu X., Qiu H. (2011). Thermal bubble dynamics under the effects of an acoustic field. Heat Transfer Eng..

[17] Takeuchi H., Motosuke M., Honami S. (2012). Noncontact bubble manipulation in microchannel by using photothermal Marangoni effect. Heat Transfer Eng..

[18] Vafaei S., Wen D., Borca-Tasciuc T. (2011). Nanofluids surface wettability through asymptotic contact angle. Langmuir.

[19] Vafaei S., Purkayastha A., Jain A., Ramanath G., Borca-Tasciuc T. (2009). The effect of nanoparticles on the liquid-gas surface tension of Bi_2_Te_3_ nanofluids. Nanotechnology.

[20] Vafaei S., Podowski M.Z. (2005). Analysis of the relationship between liquid droplet size and contact angle. Adv. Colloid Interface Sci..

[21] Ally J., Kappl M., Butt H.J., Amirfazli A. (2010). Detachment force of particles from air-liquid interfaces of films and bubbles. Langmuir.

[22] Sefiane K., Skilling J., MacGillivray J. (2008). Contact line motion and dynamic wetting of nanofluid solutions. Adv. Colloid Interface Sci..

[23] Kim S.J., Bang I.C., Buongiorno J., Hu L.H. (2007). Surface wettability change during pool boiling of nanofluids and its effect on critical heat flux. Int. J. Heat Mass Transfer.

[24] Vafaei S., Tuck C., Wildman R., Ashcroft I. (2016). Spreading of the nanofluid triple line in ink jet printed electronics tracks. Addit. Manuf..

[25] Ogino K., Tsubaki N., Abe M. (1985). Solution properties of mixed surfactant system: VI. The effect of oxyethylene groups in nonionic surfactant on surface tension of anionic-nonionic surfactant systems. J. Colloid Interface Sci..

